# Does refugee status matter? Medical needs of newly arrived asylum seekers and resettlement refugees - a retrospective observational study of diagnoses in a primary care setting

**DOI:** 10.1186/s13031-019-0223-z

**Published:** 2019-08-20

**Authors:** Evelyn Kleinert, Frank Müller, Ghefar Furaijat, Nele Hillermann, Alexandra Jablonka, Christine Happle, Anne Simmenroth

**Affiliations:** 1 Department of General Practice, University Medical Centre Göttingen / Georg-August-University, Humboldtallee 38, 37073 Göttingen, Germany; 2 Department of Clinical Immunology and Rheumatology, Hannover Medical School, Carl-Neuberg-Straße 1, 30625 Hannover, Germany; 3 German Center for Infection Research, Partner site Hanover-Brunswick, Hannover, Germany; 4 Department of Pediatrics, Neonatology, and Allergology, Hannover Medical School, Carl-Neuberg-Straße 1, 30625 Hannover, Germany; 5 Department of General Practice, University Medical Centre Würzburg, Josef-Schneider-Straße 2, 97080 Würzburg, Germany

**Keywords:** Primary healthcare, Resettlement refugees, Asylum seekers, Asylum status, Common diseases, Migrant, Infections, Mental health

## Abstract

**Background:**

Providing adequate healthcare to newly arrived refugees is considered one of the significant challenges for the German healthcare system. These refugees can be classified mainly into two groups: asylum seekers (who have applied for asylum after arrival in Germany and are waiting for the refugee-status decision) and resettlement refugees (who have already been granted asylum status before arriving in Germany). Whereas earlier studies have explored the health status of asylum seekers especially in terms of mental and behavioural disorders and infectious diseases without distinguishing between these two groups, our study aims to evaluate possible relationships of asylum status and medical needs of these two groups with a special focus on mental and behavioural disorders and infectious diseases.

**Methods:**

In this retrospective observational study, collected data on all asylum-seeker and resettlement-refugee patients (*N* = 2252) of a German reception centre (August 2017 to August 2018) is analysed by absolute and relative frequencies and medians. Patient data, collected by chart review, include age, gender, country of origin, asylum status, and diagnoses (ICD-10). To describe the relationship between sociodemographic factors (including asylum status) and diagnoses, we used tests of significance and bivariate correlations with Spearman correlation coefficients. All collected data are pseudonymised.

**Results:**

Of all 2252 patients, 43% were resettlement refugees. In almost all ICD-10 categories, asylum seekers received significantly more diagnoses than resettlement refugees. According to our data, asylum seekers presented with mental and behavioural disorders nine times more often (9%) than resettlement refugees (1%). In the case of infectious diseases, the results are mixed: asylum seekers were twice as frequently (11%) diagnosed with certain infectious and parasitic diseases than resettlement refugees (5%), but resettlement refugees were treated twice as often (22% of the asylum seekers and 41% of the resettlement refugees) for diseases of the respiratory system, of which 84% were acute respiratory infections (in both groups).

**Conclusion:**

This study indicates that patients with unregulated migration more frequently present symptoms of psychiatric diseases and somatoform symptoms than resettlement refugees. A health policy approach within migration policy should aim to enable persecuted persons to migrate under regulated and safe conditions.

**Trial registration:**

German Clinical Trials Register: DRKS00013076, retrospectively registered on 29.09.2017.

**Electronic supplementary material:**

The online version of this article (10.1186/s13031-019-0223-z) contains supplementary material, which is available to authorized users.

## Background

As a result of the ongoing global migrant and refugee crisis, medical conditions and needs of recently arrived refugees are an increasingly frequent subject of research [1–4], especially in terms of infectious diseases and mental health issues. For infectious diseases, studies verify partly poor vaccination conditions and high rates of bacterial and viral respiratory system infections among newly arrived refugees [5–8]. Publications regarding mental health issues in their turn show higher rates of anxiety, depression, traumatisation, and post-traumatic stress disorder, as well as symptoms of somatisation [9, 10].

The relationship between refugee or asylum conditions on the one hand and the medical needs of refugees on the other has not been scientifically studied so far. Nonetheless, evidence suggests that conditions related to escape, such as experiences of uncertainty and lack of control over current/future life, strongly influence the health status of affected people and place them at high risk for psychosocial distress [11]. During a long migration process, overcrowding, inadequate water supply, malnutrition, poor sanitation, and physical and psychological stress also predispose refugees to several infectious diseases [2, 7]. Riccardo et al. (2015) identified migration-specific risks for infectious diseases, such as country of origin and migration trajectory, and migration-specific health access barriers that differ according to the migration status [12]. Based on this background, we were interested to determine whether a correlation between escape conditions and medical needs of newly arrived refugees could be found.

Refugee status is a very strong indicator of escape conditions. The United Nations High Commission for Refugees (UNHCR) distinguishes between “internally displaced people” (40 million worldwide who fled within their home country), “refugees” (25.4 million worldwide who fled outside their home country) and “asylum-seekers” (3.1 million worldwide whose request for sanctuary has yet to be processed). Eighty-five percent of the world’s displaced people are hosted in developing countries. The top refugee-hosting countries are Turkey (3 million refugees), Uganda (1.4 million refugees), Pakistan (1.4 million refugees), Lebanon (1 million refugees), and Iran (979,400 refugees) [13]. A subgroup of the 25.4 million refugees is the group of 102,800 resettled refugees. These resettlement refugees have no prospects of becoming integrated into the first country they entered as refugees and are unable to return to their home countries. The UNHCR resettlement programme intends to permanently redistribute refugees from third countries for purposes of humanitarian reception. At their arrival in the new hosting country, resettlement refugees have already gone through the UNHCR refugee-status determination process and therefore do not need to apply for asylum there. In addition to Germany, other European countries (e.g. the United Kingdom, Sweden, and France) as well as the United States, Canada, and Australia take part in the UNHCR resettlement programme.

The UNHCR defines the following categories for resettlement submission: legal and/or physical protection, medical needs (in particular, life-saving treatment that is unavailable in the initially receiving country), women and girls at risk, family reunification, children and adolescents at risk, and lack of foreseeable alternative durable solutions [14].

Displaced people who have legal residence status in western industrialised nations can be divided into two groups: asylum seekers, and resettlement refugees. These two groups differ significantly in their escape conditions and their legal status [15]. In addition to these two groups, there are refugees without legal residence status. Due to the lack of registration, no valid data is available for this group. Therefore, they are not taken into account here.

### Asylum seekers and resettlement refugees in Germany

In Germany, most refugees arrive as asylum seekers after having followed an individual escape route through several countries. They apply for asylum recognised under the German asylum law or the Geneva Convention [16]. Almost 200,000 asylum applications were submitted in 2017 and about 150,000 in 2018. Most asylum seekers came from Syria (25%), Iraq (11%), Afghanistan (8%), and Eritrea (5%) [17]. Asylum seekers are initially housed in refugee camps in various locations in Germany (usually several weeks to months) before they are assigned to other dormitories or move to individual apartments with supervision and support of the social services.

The number of resettlement refugees is much smaller than that of asylum seekers: 3867 resettlement refugees (mainly of Syrian nationality) arrived in Germany in 2017 [18], of which 2988 came from refugee camps in Lebanon and Turkey [19]. In addition to UNHCR submission categories, resettlement refugees who wish to come to Germany must also meet at least one of the following criteria: preservation of the integrity of the family unit; family or other ties to Germany that promote integration; the ability to become integrated (e.g. the level of schooling/vocational training received, work experience, knowledge of the language); or a degree of vulnerability [20–22].

With the exception of people with medical conditions that are so serious that they need immediate hospital treatment, all resettlement refugees in Germany are first sent to the transit camp in Friedland, Lower Saxony. Here they are given initial admission forms, and after a welcoming course, they are distributed to other German Federal States [22].

Safe escape routes have for a long time been part of the political demands because they can contribute significantly to the physical and mental state of health of refugees. In addition, medical needs (in particular, life-saving treatment that is unavailable in the initially receiving country) are part of the five categories for resettlement submission of the UNHCR.

Regarding the conditions of escape and residence, Table 1 shows the main differences between asylum seekers and resettlement refugees. These differences may result in greater physical and psychological stress that asylum seekers suffer compared to resettlement refugees. Our hypothesis is that these differences indicate that asylum seekers have higher medical needs, particularly in regard to infectious diseases, mental and behavioural disorders.

**Table 1:** Conditions of escape and residence based on the asylum status in Germany

	Asylum seekers	Resettlement refugees
Journey to Germany	Possible long escape through different countries with possible malnutrition, inadequate water supply, lack of sleep, poor sanitation, experiences of violence during their journey	Organised safe trip to Germany from a third country (e.g. Lebanon or Turkey)
Legal situation in Germany	Approval of asylum application is uncertain, long waiting times, fear of deportation, restricted access to the labour market that depends on the decision of the asylum procedure	Residence permit for 2 years with good perspective to obtain a permanent residence permit; work permit after arrival at the final place of residence
Length of stay in Friedland transit camp	3 to 12 weeks	About 2 weeks
Access to health care before entering Germany	Restricted, if any; access to medical care in most cases	Basic medical care in the third country is provided (including standard vaccinations)
Access to health care in Germany	Restricted for the first 15 months	Restricted only during their accommodation in Friedland transit camp

The aim of this study therefore is to project the possible medical needs of asylum seekers and resettlement refugees by means of their utilization of health services resulting in the medical diagnoses given by the general practitioners. The focus is on mental and behavioural disorders and infectious diseases through analysis of primary care medical charts.

## Methods

### Setting

The transit camp in Friedland was founded in 1945 in the middle of Germany at the former border between East and West Germany in Lower Saxony. Since its foundation, it has accommodated over 4 million people arriving in the Federal Republic of Germany [10]. The camp has a capacity of 1000 beds and is operated by about 100 employees. The transit camp has a primary healthcare centre, which is run by six part-time general practitioners (GPs) and nurses. Consultation hours are Monday through Friday for 2–3 h. Asylum seekers and resettlement refugees who are housed in Friedland have to visit during these consultation hours for any type of medical care, except in case of emergency. This enabled us to record an almost full survey of all resettlement refugees coming to Germany and additionally all asylum seekers housed in Friedland. To minimise the influence of the different lengths of stay of asylum seekers and resettlement refugees, we calculated a weighting factor of 1.86 for resettlement refugees based on person days (number of attendance days in the camp of resettlement refugees (*N* = 39,347) and asylum seekers (*N* = 73,532)). This weighting was used for all further analyses, except for sociodemographic data.

### Data management

We here analyse data of all 2252 resettlement-refugee and asylum-seeker patients who visited the primary healthcare centre in Friedland between 15 August 2017 and 15 August 2018 (366 days). The patient data were collected by chart review and included age, gender, country of origin, asylum status, and diagnoses. All collected data are pseudonymised. A detailed description of the project was published elsewhere [23]. All diagnoses and symptoms were coded according to the International Classification of Diseases (ICD-10). The results were converted into dichotomous variables. Additionally, a new variable “somatoform symptoms” was calculated. This variable includes diffuse symptoms such as headaches, nausea, dizziness, or insomnia (complete presentation can be found in Additional file 1) if they were not associated with any other disease explaining the symptom. The variable “somatoform symptoms” serves to supplement the diagnoses of mental and behavioural disorders (F00-F99). These diagnoses can be difficult to assess due to the short duration of treatment and severe language barriers. Some symptoms must persist for a certain period of time in order to be diagnosed (e.g., symptoms as lowering of mood, reduction of energy, or decrease in activity must be present for at least 2 weeks to diagnose depression). Therefore, it can be assumed that residents of initial reception facilities are underdiagnosed regarding mental and behavioural disorders.

Infectious diseases were recorded using ICD-10 category A00-B99, that is, certain infectious and parasitic diseases. Management of infectious respiratory diseases in primary care does not depend mainly on laboratory results but on clinical presentation, therefore we also took acute diagnoses of respiratory diseases (J00-J22) into account. The five most common diagnoses of asylum seekers and resettlement refugees within all ICD-10 chapters are shown in Additional file 2.

The statistical software package IBM SPSS statistics 25 was used for all analyses. Sociodemographics were described by absolute and relative frequencies and by medians. For a group comparison of diagnoses, we split the sample into the two groups of refugees (asylum seekers and resettlement refugees). Differences between the two groups were tested using Fisher’s exact test. For differences in age, the Mann-Whitney U test was used. The influence of age, gender, and asylum status on diagnoses and prescriptions was measured using bivariate correlations with Spearman correlation coefficients.

## Results

Within the observed time, a total of 5206 people (2140 asylum seekers (41%) and 3066 resettlement refugees (59%)) were temporarily accommodated in the transit camp in Friedland. Of these 5206 attendees, 2252 persons (43%) had at least one regular consultation at Friedland primary healthcare centre. Therefore, 1293 (60%) of all present asylum seekers and 959 (30%) of all present resettlement refugees are regarded as patients. Fifty-one percent of asylum-seeker patients and 49% of resettlement-refugee patients were female.

**Fig. 1:**
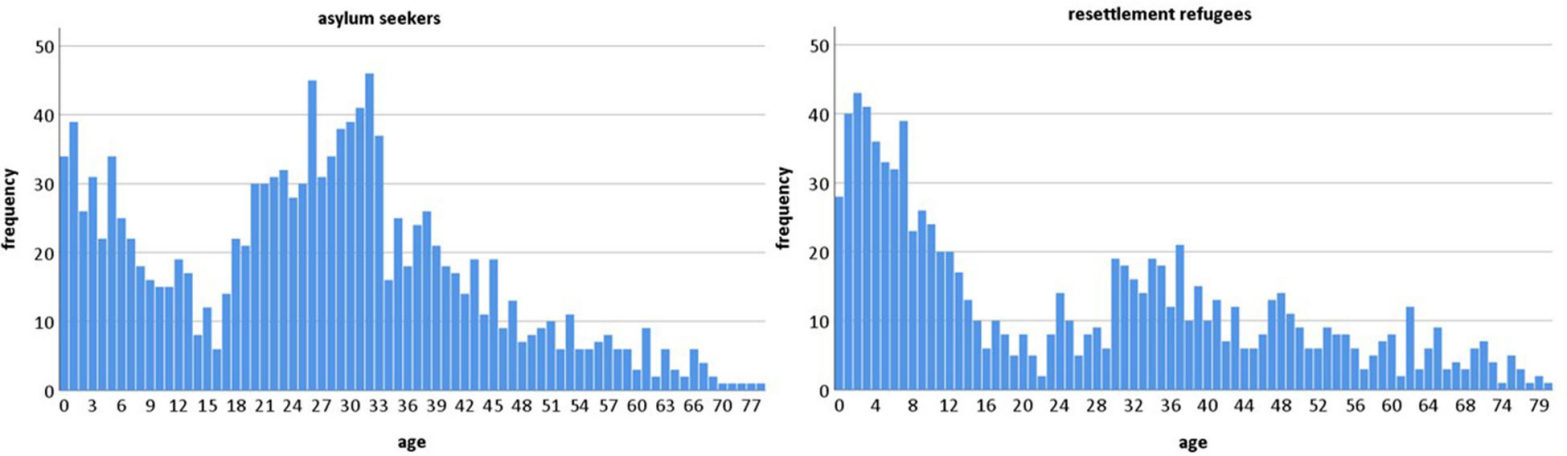
Age distribution of asylum seekers and resettlement refugees

Asylum-seeker patients mostly came from Iraq (17%), Syria (11%), Georgia (11%), Afghanistan (10%), and Iran (8%). For resettlement-refugee patients, the country in which they had applied for resettlement was registered instead of their country of origin. At least 81% of these patients were Syrians. The median age of all patients was 26 years, SD 18.529 (asylum seekers: 27 years, range 0–79; resettlement refugees: 20 years, range 0–81, *p* = 0.001). Figure 1 shows that there are more children than adults among the resettlement refugees. Twenty-nine percent of asylum seekers and 48% of resettlement refugees were younger than 18 years.

### Most frequent symptoms of illness in medical consultations

**Fig. 2:**
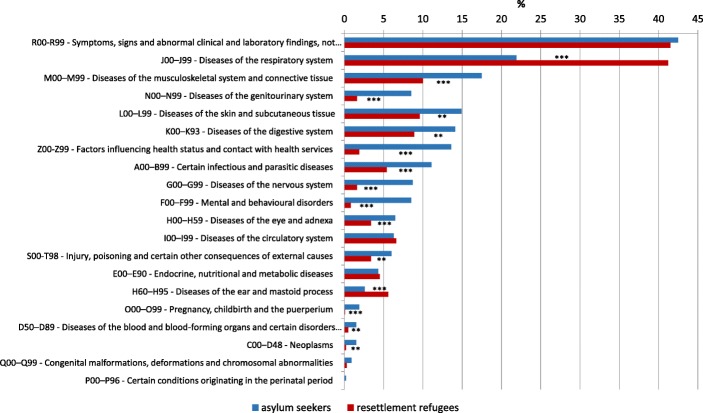
Percentage of asylum-seeker and resettlement-refugee patients presenting with different symptoms and diagnoses according to ICD-10. * *p* ≤ 0.05. ** *p* ≤ 0.01. *** *p* ≤ 0.001

Of all 2252 patients, 2054 patients (91.2%) received at least one diagnosis according to ICD-10. Figure 2 shows that 43% of asylum-seeker patients and 42% of resettlement-refugee patients presented diagnoses in the category R00-R99, which includes symptoms, signs, abnormal clinical and laboratory findings, and ill-defined conditions for which no diagnosis classifiable elsewhere is recorded. Most common diagnoses in this category are cough, headache, sore throat, fever and abdominal pain. These unspecific codes are very common in general practice in Germany. Other common reasons for consultation are diseases of the respiratory system (22% of asylum seekers, 41% of resettlement refugees) and diseases of the musculoskeletal system and connective tissue (18% of asylum seekers and 10% of resettlement refugees). Considering only adult patients, 42% of asylum seekers and 35% of resettlement refugees presented symptoms, signs, and abnormal clinical and laboratory findings in the category R00-R99 (*p* ≤ 0.01), and 14% of asylum seekers and 28% of resettlement refugees had diseases of the respiratory system (*p* ≤ 0.001). Diseases of the musculoskeletal system were represented slightly more frequently than in the overall sample, with 23% for asylum seekers and 19% for resettlement refugees (*p* ≤ 0.05). In total, asylum seekers were diagnosed more frequently in almost all diagnostic categories. Resettlement refugees, on the other hand, were only more frequently affected by respiratory and ear diseases. This difference also persists when only adult patients are considered.

### Mental and behavioural disorders

Regarding mental and behavioural disorders, Fig. 2 shows that 9% of asylum seekers and 1% of resettlement refugees have received diagnoses in the category F00-F99 (*p* ≤ 0.001). This effect increases when only adult patients are considered (11% of asylum seekers, and 1% of resettlement refugees). The most frequent diagnoses were depressive episodes, reaction to severe stress, and adjustment disorders as well as other anxiety disorders (see Table 2). In addition, 15% of asylum seekers (*N* = 194) and 7% of resettlement refugees (*N* = 117) presented somatoform symptoms (*p* ≤ 0.001).

**Table 2:** Most frequent diagnoses related to mental and behavioural disorders (multiple diagnoses per patient possible); weighted

	Asylum seekers	N	Resettlement refugees	N
F00-F99 Mental and behavioural disorders	F32 - Depressive episode	95	F89 - Unspecified disorder of psychological development	9
	F43 - Reaction to severe stress, and adjustment disorders	61	F32 - Depressive episode	4
	F41 - Other anxiety disorders	17	F41 - Other anxiety disorders	2
	F99 - Mental disorder, not otherwise specified	8	F79 - Unspecified mental retardation	2
	F44 - Dissociative [conversion] disorders	5	others	0
	others	31		
Somatoform symptoms		265		128

In summary, 24% of asylum seekers (*N* = 304) and 7% of resettlement refugees (*N* = 132) sought medical help for mental health problems.

### Infectious diseases

To detect infectious diseases, we used the ICD-10 category certain infectious and parasitic diseases (A00-B99). As shown in Fig. 2, 11% of asylum seekers and 5% of resettlement refugees have received diagnoses from this category (*p* ≤ 0.001), with gastroenteritis and colitis, scabies, and chronic viral hepatitis as the most frequent diagnoses (see Table 3).

**Table 3:** Most frequent diagnoses related to certain infectious and parasitic diseases (multiple diagnoses per patient possible); weighted

	Asylum seekers	N	Resettlement refugees	N
A00-B99 Certain infectious and parasitic diseases	B86 - Scabies	35	A09 - Gastroenteritis and colitis of unspecified origin	52
	B18 - Chronic viral hepatitis C	34	B86 - Scabies	7
	A09 - Gastroenteritis and colitis of unspecified origin	23	B37 - Candidal stomatitis	7
	B83 - Visceral larva migrans	19	B01 - Varicella without complication	7
	B00 - Herpesviral vesicular dermatitis	17	B00 - Herpesviral vesicular dermatitis	6
	others	75	others	30

The relation between asylum seekers and resettlement refugees is reversed in the diagnoses of respiratory diseases: 22% of asylum seekers and 41% of resettlement refugees received such a diagnosis (see Fig. 2).

In both groups, 84% of all “J-diagnoses” concern acute infectious diseases (J00-J22). Most common are acute upper respiratory infections, followed by other acute lower respiratory infections (see Table 4).

**Table 4:** Number of infectious diseases within respiratory diseases (multiple diagnoses per patient possible); weighted

	Asylum seekers (N)	Resettlement refugees (N)
J00-J06 Acute upper respiratory infections	281	319
J09-J18 Influenza and pneumonia	4	3
J20-J22 Other acute lower respiratory infections	60	90
Other respiratory diseases	67	77

### Sociodemographic factors influencing mental illnesses and infectious diseases

Regarding the hypothesis that asylum seekers have higher medical needs in relation to treatments for mental and behavioural disorders and infectious diseases, Table 5 shows bivariate correlations for these diagnoses and symptoms. Accordingly, there are slightly negative correlations between asylum status and mental and behavioural disorders, and somatoform symptoms as well as certain infectious and parasitic diseases. This supports the hypothesis that asylum seekers are more frequently affected by all these diagnoses than resettlers, but resettlement refugees are more often affected by diseases of the respiratory system. However, age also has a significant influence in the sense that adult patients are more often affected by mental and behavioural disorders and somatoform symptoms. Children and adolescents younger than 18 years, on the other hand, are more frequently affected by certain infectious and parasitic diseases and diseases of the respiratory system. Gender has only a weak influence on the diagnosis of somatoform symptoms in the sense that women are slightly more likely to seek medical help with somatoform symptoms. Gender had no influence on mental and behavioural disorders and certain infectious and parasitic diseases (see Table 5).

**Table 5:** Spearman correlation coefficients for several diagnoses (0 = no diagnosis, 1 = at least one diagnosis); weighted; entire cohort

	Mental and behavioural disorders	Somatoform symptoms	Certain infectious and parasitic diseases	Diseases of the respiratory system
Age (0 = child; 1 = adult)	.100***	.163***	−.055**	−.309***
sex (1 = female, 2 = male)	−.014	−.038^*^	.008	.011
asylum status (1 = AS, 2 = RR)	−.192^***^	−.138^***^	−.104^***^	.200***

*AS* Asylum seekers, *RR* Resettlement refugees

* *p* ≤ 0.05. ** *p* ≤ 0.01. *** *p* ≤ 0.001

To preclude the influence of age, Table 6 shows bivariate correlations only on patients older than 18 years. The highly significant correlations between asylum status and diagnostic groups clearly still persist, although there are minimal shifts. In mental and behavioural disorders and certain infectious and parasitic diseases, the effect increases somewhat, whereas it decreases slightly in somatoform symptoms and diseases of the respiratory system.

**Table 6:** Spearman correlation coefficients for several diagnoses (0 = no diagnosis, 1 = at least one diagnosis); weighted, adult cohort

	Mental and behavioural disorders	Somatoform symptoms	Certain infectious and parasitic diseases	Diseases of the respiratory system
sex (1 = female, 2 = male)	−.019	−.038	.038	−.035
asylum status (1 = AS, 2 = RR)	−.214***	−.101***	−.130***	.169***

*AS* Asylum seekers, *RR* Resettlement refugees

* *p* ≤ 0.05. ** *p* ≤ 0.01. *** *p* ≤ 0.001

## Discussion

Based on the background of different conditions of escape and residence, diagnoses of newly arrived asylum seekers and resettlement refugees in an on-site primary healthcare ward in an initial reception centre were recorded. To our knowledge, this is the first survey that acquired diagnoses of almost the complete group of resettlement refugees in Germany and gives a direct comparison with asylum seekers in the same setting. We found significantly more diagnoses in the field of specific infectious diseases and especially psychiatric disorders in the group of asylum seekers. According to our data, asylum seekers presented with mental and behavioural disorders nine times more often (9%) than resettlement refugees (1%). Somatoform symptoms (e.g. headaches, nausea, unspecific abdominal pain, and insomnia) were diagnosed more frequently in both groups. This may be because a diagnosis of a mental and behavioural disorder requires more background information of the patients and a long observation period, which was not possible in this context. The limited length of stay and existing language barriers often prevent a detailed psychosocial anamnesis, especially in Friedland, where interpreters are usually rarely available during medical consultations. Under these conditions, it is difficult to diagnose a mental and behavioural disorder, and the GPs only describe somatoform symptoms. However, somatoform symptoms were also diagnosed twice as often in asylum seekers (15%) than in resettlement refugees (7%). On one hand, these results correspond to the risk factors described in the literature, such as insecurity about current life and future [11, 24], to which asylum seekers are much more exposed. Similarly, resettlement refugees most probably also experienced traumatic events, but since their trip to Germany is already organized, they travel in much better conditions and have a secure perspective, while for asylum seekers the uncertainty remains for a longer period of time. On the other hand, it is also possible that resettlement refugees do not consult a physician because they know that they will reach their final residence 2 weeks later and then will be allowed to use regular healthcare services. However, this risk of bias appears to be quite low because Wetzke et al. (2018) were able to show in a comparable initial reception centre that asylum seekers most often sought medical help during the first week and that their use of the medical services decreased with longer periods of stay [25].

These data do not straightforwardly support our hypothesis that asylum seekers suffer more frequently from infectious diseases than resettlement refugees. However, the evidence from this study shows that asylum seekers were diagnosed twice as frequently with certain infectious and parasitic diseases (e.g., scabies, hepatitis C, gastroenteritis) but only half as often for acute infectious respiratory diseases. These differences may be due to different escape conditions, like inadequate water supply, malnutrition, poor sanitation, and lack of medical care (regarding asylum seekers) [2, 7] or group accommodation and flights (regarding resettlement refugees): There is some evidence that air-conditioning systems used in airplanes might increase the incidence of upper respiratory symptoms, which could explain the higher prevalence of acute infectious respiratory diseases in resettlement refugees in our data [26–28].

Our study is limited by a lack of systematic screening regarding infectious diseases or mental and behavioral disorders, thus only refugees who presented to medical care could be included in this study. The diagnosis was mainly based on the clinical judgement of the experienced general practitioners, laboratory or standardised mental health questionnaires were rarely used. A further aspect is the retrospective study design, which made it impossible to take other influencing factors into account (e.g. physical and psychological living conditions before and during the escape journey or changes in political conditions in the countries of origin during the study period). However, the influence of age and gender of refugees were controlled on the basis of sociodemographic data, with women presenting slightly more often with somatoform symptoms than men. Age had a much greater influence than gender, especially on respiratory diseases, which affected children significantly more often than adults. Even after children were excluded, a highly significant correlation between asylum status and all diagnostic groups remains, with asylum seekers receiving diagnoses with mental and behavioural disorders, somatoform symptoms, and certain infectious and parasitic diseases more frequently than resettlement refugees, even though the effect is rather weak. Small correlations can best be identified in studies with large samples, which is a major strength of this study. Here, medical data of almost all resettlement refugees coming to Germany during their first weeks of stay are presented for the first time.

## Conclusions

The increasing population of refugees and asylum seekers poses new challenges to the healthcare systems of the receiving host countries and communities. The present study shows that asylum seekers seem to need more healthcare service than resettlement refugees in the context of an initial reception center with regard to psychological disorders and symptoms as well as specific infectious and parasitic diseases. To reduce the need for medical treatment in these settings, resettlement programs may be considered a better alternative that will enable a planned safe migration with more secure prospects, for host countries and migrants.

## Additional files

Additional file 1: Somatoform symptoms. (DOCX 13 kb)

Additional file 2: Overview diagnoses. (DOCX 21 kb)

## Data Availability

The datasets used and/or analysed during the current study are available from the corresponding author on reasonable request.

## Abbreviations

AS: Asylum Seeker
GP: General Practitioner
ICD-10: International Classification of Diseases
RR: Resettlement Refugee
UNHCR: United Nations High Commission for Refugees
