# Supplementary material for: Does refugee status matter? Medical needs of newly arrived asylum seekers and resettlement refugees - a retrospective observational study of diagnoses in a primary care setting
Source: Confl Health. 2019 Aug 20;13:39. doi: 10.1186/s13031-019-0223-z (PMC6700982; doi:10.1186/s13031-019-0223-z)
Supplement: Supplementary file 1 — Somatoform symptoms. (DOCX 13 kb) [file 13031_2019_223_MOESM1_ESM.docx]

**Additional file 1**

**Computation of the variable "somatoform symptoms”**

The following symptoms are coded as “somatoform symptoms” if they are not associated with any other disease.

| G44.2 | Tension-type headache |
| --- | --- |
| G47.0 | Disorders of initiating and maintaining sleep |
| G47.1 | Disorders of excessive somnolence |
| G47.2 | Disorders of the sleep-wake schedule |
| G47.9 | Sleep disorder, unspecified |
| H81.9 | Disorder of vestibular function, unspecified |
| K30 | Functional dyspepsia |
| K58 | Irritable bowel syndrome |
| K59.0 | Constipation |
| K59.1 | Functional diarrhoea |
| K59.8 | Other specified functional intestinal disorders |
| K59.9 | Functional intestinal disorder, unspecified |
| M54.3 | Sciatica |
| M54.4 | Lumbago with sciatica |
| M54.5 | Low back pain |
| M54.6 | Pain in thoracic spine |
| M54.8 | Other dorsalgia |
| M54.9 | Dorsalgia, unspecified |
| M79.1- | Myalgia |
| M79.6- | Pain in limb |
| M79.7- | Fibromyalgia |
| R00.2 | Palpitations |
| R00.8 | Other and unspecified abnormalities of heart beat |
| R03.1 | Nonspecific low blood-pressure reading |
| R06.6 | Hiccough |
| R06.7 | Sneezing |
| R07.1 | Chest pain on breathing |
| R10.1 | Pain localized to upper abdomen |
| R10.2 | Pelvic and perineal pain |
| R10.3 | Pain localized to other parts of lower abdomen |
| R10.4 | Other and unspecified abdominal pain |
| R11 | Nausea and vomiting |
| R14 | Flatulence and related conditions |
| R19.1 | Abnormal bowel sounds |
| R20.2 | Paraesthesia of skin |
| R20.8 | Other and unspecified disturbances of skin sensation |
| R39.1 | Other difficulties with micturition |
| R41 | Disorientation, unspecified |
| R42 | Dizziness and giddiness |
| R43 | Disturbances of smell and taste |
| R45 | Symptoms and signs involving emotional state |
| R46.2 | Strange and inexplicable behaviour |
| R46.5 | Suspiciousness and marked evasiveness |
| R46.8 | Other symptoms and signs involving appearance and behaviour |
| R49 | Voice disturbances |
| R51 | Headache |
| R52 | Pain, not elsewhere classified |
| R53 | Malaise and fatigue |
| R55 | Syncope and collapse |
| R63.0 | Anorexia |
