# Supplementary material for: Does refugee status matter? Medical needs of newly arrived asylum seekers and resettlement refugees - a retrospective observational study of diagnoses in a primary care setting
Source: Confl Health. 2019 Aug 20;13:39. doi: 10.1186/s13031-019-0223-z (PMC6700982; doi:10.1186/s13031-019-0223-z)
Supplement: Supplementary file 2 — Overview diagnoses. (DOCX 21 kb) [file 13031_2019_223_MOESM2_ESM.docx]

Additional file 2

**The five most common diagnoses of asylum seekers and resettlement refugees within all ICD-10 chapters** (multiple diagnoses per patient possible; with a weighting factor of 1.86 for resettlement refugees)

|  | **Asylum seekers** | | **Resettlement refugees** | |
| --- | --- | --- | --- | --- |
| **ICD10 group** | **ICD10** | **N** | **ICD10** | **N** |
| A00-B99  Certain infectious and parasitic diseases | B86 - Scabies | 35 | A09 - Gastroenteritis and colitis of unspecified origin | 52 |
|  | B18 Chronic viral hepatitis C | 34 | B86 - Scabies | 7 |
|  | A09 - Gastroenteritis and colitis of unspecified origin | 23 | B37 - Candidal stomatitis | 7 |
|  | B83 - Visceral larva migrans | 19 | B01 - Varicella without complication | 7 |
|  | B00 - Herpesviral vesicular dermatitis | 17 | B00 - Herpesviral vesicular dermatitis | 6 |
|  | other | 75 | other | 30 |
| C00-D48 Neoplasms | C53 - Malignant neoplasm of cervix uteri | 13 | D37 - Neoplasm of uncertain or unknown behaviour of oral cavity and digestive organs | 6 |
|  | D25 - Leiomyoma of uterus | 7 | other | 0 |
|  | D48 - Neoplasm of uncertain or unknown behaviour of other and unspecified sites | 6 |  |  |
|  | C49 - Malignant neoplasm of other connective and soft tissue | 4 |  |  |
|  | C91 - Lymphoid leukaemia | 4 |  |  |
|  | other | 11 |  |  |
| D50-D90  Diseases of the blood and blood-forming organs and certain disorders involving the immune mechanism | D50 - Iron deficiency anaemia | 11 | D56 - Thalassaemia | 9 |
|  | D64 - Other anaemias | 9 | D50 - Iron deficiency anaemia | 2 |
|  | D69 - Purpura and other haemorrhagic conditions | 7 | other | 0 |
|  | D56 - Thalassaemia | 1 |  |  |
|  | D72 - Other disorders of white blood cells | 1 |  |  |
|  | other | 1 |  |  |
| E00-E90  Endocrine, nutritional and metabolic diseases | E11 - Non-insulin-dependent diabetes mellitus | 40 | E11 - Non-insulin-dependent diabetes mellitus | 54 |
|  | E03 - Other hypothyroidism | 11 | E14 - Unspecified diabetes mellitus | 9 |
|  | E14 - Unspecified diabetes mellitus | 11 | E78 - Disorders of lipoprotein metabolism and other lipidaemias | 9 |
|  | E04 - Other nontoxic goitre | 9 | E66 - Obesity | 9 |
|  | E78 - Disorders of lipoprotein metabolism and other lipidaemias | 8 | E03 - Other hypothyroidism | 4 |
|  | other | 22 | other | 11 |
| F00-F99  Mental and behavioural disorders | F32 - Depressive episode | 95 | F89 - Unspecified disorder of psychological development | 9 |
|  | F43 - Reaction to severe stress, and adjustment disorders | 61 | F32 - Depressive episode | 4 |
|  | F41 - Other anxiety disorders | 17 | F41 - Other anxiety disorders | 2 |
|  | F99 - Mental disorder, not otherwise specified | 8 | F79 - Unspecified mental retardation | 2 |
|  | F44 - Dissociative [conversion] disorders | 5 | other | 0 |
|  | other | 31 |  |  |
| G00-G99  Diseases of the nervous system | G47 - Disorders of initiating and maintaining sleep [insomnias] | 84 | G58 - Other mononeuropathies | 6 |
|  | G40 - Epilepsy | 14 | G81 - Hemiplegia | 6 |
|  | G43 - Migraine | 14 | G54 - Nerve root and plexus disorders | 6 |
|  | G58 - Other mononeuropathies | 9 | G80 - Cerebral palsy | 4 |
|  | G80 - Cerebral palsy | 5 | G47 - Disorders of initiating and maintaining sleep [insomnias] | 2 |
|  | other | 13 | other | 7 |
| H00-H59  Diseases of the eye and adnexa | H10 - Conjunctivitis | 39 | H10 - Conjunctivitis | 39 |
|  | H53 - Visual disturbances | 29 | H57 - - Other disorders of eye and adnexa | 11 |
|  | H57 - Other disorders of eye and adnexa | 19 | H53 - Visual disturbances | 6 |
|  | H01 - Other inflammation of eyelid | 4 | H01 - Other inflammation of eyelid | 2 |
|  | H04 - Disorders of lacrimal system | 4 | H00 - Hordeolum and other deep inflammation of eyelid | 2 |
|  | other | 14 | other | 6 |
| H60-H95  Diseases of the ear and mastoid process | H66 - Suppurative and unspecified otitis media | 17 | H92 - Otalgia and effusion of ear | 50 |
|  | H92 - Otalgia and effusion of ear | 14 | H66 - Suppurative and unspecified otitis media | 47 |
|  | H60 - Otitis externa | 4 | H68 - Eustachian salpingitis and obstruction | 6 |
|  | H91 - Other hearing loss | 3 | H93 - Other disorders of ear, not elsewhere classified | 4 |
|  | H93 - Other disorders of ear, not elsewhere classified | 2 | H91 - Other hearing loss | 2 |
|  | other | 1 | other | 2 |
| I00-I99  Diseases of the circulatory system | I10 - Essential (primary) hypertension | 102 | I10 - Essential (primary) hypertension | 128 |
|  | I25 - Chronic ischaemic heart disease | 22 | I25 - Chronic ischaemic heart disease | 32 |
|  | I95 - Hypotension | 8 | I83 - Varicose veins of lower extremities | 2 |
|  | I49 - Other cardiac arrhythmias | 7 | I20 - Angina pectoris | 2 |
|  | I83 - Varicose veins of lower extremities | 3 | I50 - Congestive heart failure | 2 |
|  | other | 15 | other | 4 |
| J00-J99  Diseases of the respiratory system | J00 - Acute nasopharyngitis [common cold] | 119 | J06 - Acute upper respiratory infections of multiple and unspecified sites | 231 |
|  | J06 - Acute upper respiratory infections of multiple and unspecified sites | 86 | J00 - Acute nasopharyngitis [common cold] | 184 |
|  | J03 - Acute tonsillitis | 66 | J03 - Acute tonsillitis | 171 |
|  | J20 - Acute bronchitis | 58 | J20 - Acute bronchitis | 167 |
|  | J98 - Diseases of bronchus, not elsewhere classified | 14 | J31 - Chronic rhinitis, nasopharyngitis and pharyngitis | 47 |
|  | other | 64 | other | 112 |
| K00-K93  Diseases of the digestive system | K08 - Other disorders of teeth and supporting structures | 94 | K08 - Other disorders of teeth and supporting structures | 56 |
|  | K29 - Gastritis and duodenitis | 52 | K59 - Other functional intestinal disorders | 37 |
|  | K59 - Other functional intestinal disorders | 23 | K29 - Gastritis and duodenitis | 32 |
|  | K02 - Dental caries | 20 | K02 - Dental caries | 15 |
|  | K64 - Haemorrhoids and perianal venous thrombosis | 14 | K05 - Gingivitis and periodontal diseases | 9 |
|  | other | 58 | other | 37 |
| L00-L99  Diseases of the skin and subcutaneous tissue | L30 - Other dermatitis | 66 | L30 - Other dermatitis | 60 |
|  | L29 - Pruritus | 56 | L29 - Pruritus | 28 |
|  | L22 - Diaper [napkin] dermatitis | 28 | L23 - Allergic contact dermatitis | 15 |
|  | L02 - Hautabszess, Furunkel und Karbunkel | 24 | L22 - Diaper [napkin] dermatitis | 9 |
|  | L70 - Akne | 24 | L02 - Cutaneous abscess, furuncle and carbuncle | 7 |
|  | other | 116 | other | 69 |
| M00-M99  Diseases of the musculoskeletal system and connective tissue | M54 - Dorsalgia | 128 | M54 - Dorsalgia | 82 |
|  | M79 - Other soft tissue disorders, not elsewhere classified | 74 | M79 - Other soft tissue disorders, not elsewhere classified | 48 |
|  | M25 - Other joint disorders, not elsewhere classified | 71 | M25 - Other joint disorders, not elsewhere classified | 35 |
|  | M53 - Other dorsopathies, not elsewhere classified | 17 | M53 - Other dorsopathies, not elsewhere classified | 9 |
|  | M62 - Other disorders of muscle | 9 | M17 - Gonarthrosis [arthrosis of knee] | 9 |
|  | other | 51 | other | 13 |
| N00-N99  Diseases of the genitourinary system | N39 - Other disorders of urinary system | 20 | N39 - Other disorders of urinary system | 20 |
|  | N94 - Pain and other conditions associated with female genital organs and menstrual cycle | 20 | N92 - Excessive, frequent and irregular menstruation | 4 |
|  | N30 - Cystitis | 16 | N30 - Cystitis | 2 |
|  | N92 - Excessive, frequent and irregular menstruation | 14 | N93 - Other abnormal uterine and vaginal bleeding | 2 |
|  | N89 - Other noninflammatory disorders of vagina | 12 | N64 - Other disorders of breast | 2 |
|  | other | 58 | other | 2 |
| O00-O99  Pregnancy, childbirth and the puerperium | O26 - Maternal care for other conditions predominantly related to pregnancy | 7 | O36 - Maternal care for other known or suspected fetal problems | 2 |
|  | O82 - Single delivery by caesarean section | 7 | other | 0 |
|  | O99 - Anaemia complicating pregnancy, childbirth and the puerperium | 7 |  |  |
|  | O24 - Diabetes mellitus in pregnancy | 6 |  |  |
|  | O92 - Other disorders of breast and lactation associated with childbirth | 3 |  |  |
|  | other | 7 |  |  |
| P00-P96  Certain conditions originating in the perinatal period | P38 - Omphalitis of newborn with or without mild haemorrhage | 1 |  |  |
|  | P92 Feeding problems of newborn | 1 |  |  |
|  | other | 0 | other | 0 |
| Q00-Q99  Congenital malformations, deformations and chromosomal abnormalities | Q66 - Congenital deformities of feet | 3 | Q74 - Other congenital malformations of limb(s) | 2 |
|  | Q37 - Cleft palate with cleft lip | 2 | Q66 - Congenital deformities of feet | 2 |
|  | Q65 - Congenital deformities of hip | 2 | other | 0 |
|  | Q67 - Congenital musculoskeletal deformities of head, face, spine and chest | 2 |  |  |
|  | Q05 - Spina bifida | 1 |  |  |
|  | other | 4 |  |  |
| R00-R99  Symptoms, signs and abnormal clinical and laboratory findings, not elsewhere classified | R10 - Abdominal and pelvic pain | 190 | R05 - Cough | 260 |
|  | R51 - Headache | 164 | R07 - Pain in throat and chest | 242 |
|  | R05 - Cough | 145 | R50 - Fever of other and unknown origin | 212 |
|  | R07 - Pain in throat and chest | 120 | R51 - Headache | 99 |
|  | R50 - Fever of other and unknown origin | 78 | R10 - Abdominal and pelvic pain | 76 |
|  | other | 308 | other | 188 |
| S00-T98  Injury, poisoning and certain other consequences of external causes | T14 - Injury of unspecified body region | 41 | T14 - Injury of unspecified body region | 11 |
|  | T78 - Adverse effects, not elsewhere classified | 11 | T78 - Adverse effects, not elsewhere classified | 9 |
|  | S80 - Superficial injury of lower leg | 7 | T30 - Burn and corrosion, body region unspecified | 9 |
|  | S01 - Open wound of head | 4 | S69 - Other and unspecified injuries of wrist and hand | 9 |
|  | T30 - Burn and corrosion, body region unspecified | 4 | S01 - Open wound of head | 4 |
|  | other | 33 | other | 26 |
| V01-Y84  External causes of morbidity and mortality | - | - | - | - |
| Z00–Z99  Factors influencing health status and contact with health services | Z32 - Pregnancy examination and test | 98 | Z32 - Pregnancy examination and test | 22 |
|  | Z26 - Need for immunization against other single infectious diseases | 33 | Z20 - Contact with and exposure to communicable diseases | 7 |
|  | Z48 - Other surgical follow-up care | 24 | Z48 - Other surgical follow-up care | 4 |
|  | Z94 - Transplanted organ and tissue status | 20 | Z26 - Need for immunization against other single infectious diseases | 2 |
|  | Z27 - Need for immunization against combinations of infectious diseases | 16 | Z87 - Personal history of other diseases and conditions | 2 |
|  | other | 72 | other | 2 |
